# Assessment of disease-severity scoring systems for patients with sepsis in general internal medicine departments

**DOI:** 10.1186/cc10102

**Published:** 2011-03-14

**Authors:** Nesrin O Ghanem-Zoubi, Moshe Vardi, Arie Laor, Gabriel Weber, Haim Bitterman

**Affiliations:** 1 Carmel Medical Center. The Ruth and Bruce Rappaport Faculty of Medicine. Technion - Israel Institute of Technology, Technion City, Haifa 32000, Israel

## Abstract

**Introduction:**

Due to the increasing burden on hospital systems, most elderly patients with non-surgical sepsis are admitted to general internal medicine departments. Disease-severity scoring systems are used for stratification of patients for utilization management, performance assessment, and clinical research. Some widely used scoring systems for septic patients are inappropriate when rating non-surgical patients in a non-intensive care unit (ICU) environment mainly because their calculations require types of data that are frequently unavailable. This study aimed to assess the fitness of four scoring systems for septic patients hospitalized in general internal medicine departments: modified early warning score (MEWS), simple clinical score (SCS), mortality in emergency department sepsis (MEDS) score, and rapid emergency medicine score (REMS).

**Methods:**

We prospectively collected computerized data of septic patients admitted to general internal medicine departments in our community-based university hospital. We followed 28-day in-hospital mortality, overall in-hospital mortality, and 30- and 60-day mortality. Using a logistic regression procedure we calculated the area under ROC curve (AUC) for every scoring system.

**Results:**

Between February 1^st^, 2008 and April 30^th^, 2009 we gathered data of 1,072 patients meeting sepsis criteria on admission to general internal medicine departments. The 28-day mortality was 19.4%. The AUC for the MEWS was 0.65-0.70, for the SCS 0.76-0.79, for the MEDS 0.73-0.75, and for the REMS, 0.74-0.79. Using Hosmer-Lemeshow statistics, a lack of fit was found for the MEDS model. All scoring systems performed better than calculations based on sepsis severity.

**Conclusions:**

The SCS and REMS are the most appropriate clinical scores to predict the mortality of patients with sepsis in general internal medicine departments.

## Introduction

Sepsis is a prevalent, serious and resource-consuming medical condition. The incidence of sepsis increased by 8.7% annually from 1979 to 2000 [1]. Sepsis is the 10th leading cause of overall death in the USA, and the sixth when including pneumonia and influenza [2]. Although the in-hospital mortality rate from sepsis decreased from 27.8% (reported for the period from 1979 to 1984) to 17.9% (reported for 1995 to 2000), the absolute number of deaths increased due to its increasing incidence [1]. The markedly increased incidence resulted in an estimated US$16.7 billion annual cost related to severe sepsis in the USA [3].

Due to limited ICU resources, most septic patients, including patients with severe sepsis, are currently admitted to general departments [4,5]. The aging of Western populations is an important contributing factor to the increasing incidence of sepsis in recent years, because older people are more prone to infections. All in all, elderly patients with sepsis occupy an increasing proportion of hospital beds in general internal medicine departments.

In 2004, critical care and infectious disease experts developed management guidelines for severe sepsis and septic shock that were updated in 2008 under the auspices of the Surviving Sepsis Campaign (SSC) [6,7]. The SSC aimed to reduce mortality from sepsis via a multi-point strategy, primarily by building awareness, improving diagnosis and increasing the use of appropriate treatment. In this respect, there is a growing need for appropriate tools to assess severity of sepsis, and to enable the early detection of complex cases that warrant particular attention with rapid and appropriate treatment.

Many disease severity scoring systems related to sepsis have been developed over the years [8-12]. Most methods were devised for assessment of patients with sepsis who underwent surgery and were admitted to the ICU [13], or for specific infectious conditions (e.g., bacteremia, pneumonia) [10,12]. These classifications may not be appropriate for patients with sepsis who are being admitted to general internal medicine departments.

Only a few scoring systems exist that are not restricted to a specific medical condition or ICU setting. The Modified Early Warning Score (MEWS) is a simple physiological scoring system suitable for bedside application that was validated in a prospective cohort study on 709 medical emergency admissions. It is not a disease-specific score. It was found that a MEWS of more than four predicts increased risk of mortality with an odds ratio (OR) of 5.4. The area under the curve (AUC) for predicting 60-day mortality was 0.67 [14]. The Simple Clinical Score (SCS) was developed and validated on 9,964 patients admitted as acute medical emergencies. It is also not disease-specific. The SCS receiver operating characteristics (ROC) curve for 30-day mortality had an AUC of between 0.85 and 0.9 [15]. The Mortality in Emergency Department Sepsis (MEDS) score was developed for use with patients "at risk for infection". The study included 3,179 surgical and medical patients. It was found to predict 28-day in-hospital mortality with an AUC of 0.82 and 0.78 for derivation and validation groups, respectively [16]. The Rapid Emergency Medicine Score (REMS) was developed in non-surgical adults admitted to emergency departments over a period of one year. The AUC for predicting in-hospital mortality was found to be 0.85 [17].

The current study aimed to prospectively compare the prognostic value of these four general scoring systems in patients with sepsis upon admission to general internal medicine departments.

## Materials and methods

### Patients

The study included consecutive patients admitted to a 110-bed general internal medicine department from 1 February 2008 to 30 April 2009 in a 450-bed community-based university hospital in Haifa, Israel. All patients were over 18 years of age, and had a presumed diagnosis compatible with sepsis. The patients were identified automatically using the definition of sepsis given by the American College of Chest Physicians (ACCP)/Society of Critical Care Medicine (SCCM) Consensus Conference in 1991 [18], that is, any patient admitted with suspected infection and at least two of the criteria of Systemic Inflammatory Response Syndrome (SIRS): (1) a temperature of more than 38°C or less than 36°C; (2) an elevated heart rate greater than 90 beats per minute; (3) tachypnea, manifested by a respiratory rate greater than 20 breaths per minute or hyperventilation, as indicated by a partial pressure of arterial carbon dioxide of less than 32 mmHg; (4) an alteration in the white blood cell count, such as a count greater than 12,000/cu mm, a count less than 4,000/cu mm; or the presence of more than 10% immature neutrophils. No exclusion criteria were employed.

### Data collection

We developed a computerized database that was incorporated into our electronic medical record (EMR) system. The computerized system identified patients with presumed sepsis according to the criteria listed above. Thereafter, the physicians were instructed to input the required data necessary for the examined scoring systems (Table 1) via a mandatory questionnaire that included structured input of data, alongside automatic data gathering.

**Table 1:** Parameters required for the calculations of the examined scores

	MEWS	REMS	MEDS	SCS
Age		X	X	X
Nursing home resident			X	X
Mental status			X	X
Functional status				X
Terminal illness			X	
Diabetes (type I or II)				X
Lower respiratory tract infection			X	
New stroke on presentation				X
Heart rate	X	X		X
Temperature	X	X		X
Systolic blood pressure	X			X
Mean arterial pressure		X		
Respiratory rate	X	X	X	X
Peripheral oxygen saturation		X	X	X
AVPU score	X			
Glasgow coma scale		X		
Coma without intoxication or overdose				X
Breathless on presentation				X
Septic shock			X	
Abnormal ECG				X
Platelet count			X	
Percent bands in differential count			X	

AVPU, alert, responding to voice, responding to pain, unconscious; MEDS, mortality in emergency medicine sepsis score; MEWS, modified early warning score; REMS, rapid emergency medicine score; SCS, simple clinical score.

Data related to current illness included vital signs, such as heart rate, respiratory rate, body temperature, systolic and diastolic blood pressures, and oxygen saturation. The mean arterial pressure was calculated. Physicians were instructed to register the worst value until admission to the department, including emergency department values. Other findings at presentation included breathlessness, new stroke, intoxication or overdose, lower respiratory tract infection, AVPU (Alert, responding to Voice, responding to Pain, Unconscious) score, Glasgow coma scale score, abnormal electrocardiogram recording (other than sinus tachycardia or bradycardia), as well as clinical staging of sepsis (sepsis, severe sepsis, septic shock) based on ACCP/SCCM definitions.

Demographics and co-morbidities were also gathered: age, functional status, unable to stand unaided prior to current illness, bedridden, type of residence, mental status, diabetes mellitus, and terminal illness. Laboratory results required for scoring calculations were drawn automatically and included platelet count and band percentage in the differential count.

### Follow up

We recorded follow up survival rates for at least 60 days. For patients who died, we recorded whether the death occurred in hospital or after discharge. For every examined scoring system, we checked its fitness to predict overall death rates in different intervals of time (1, 5, 10, 30 and 60 days), as well as 28 days and overall in-hospital mortality rates. For post-discharge death, data were extracted from our EMR system, which is supplied with death data from the Ministry of Interior records.

### Statistical analysis

We described the distribution of our study group by calculating mean, range, and standard deviation. For categorical variables we calculated the distribution and cumulative distribution.

For determining the quality of the examined scoring systems we used logistic regression. For each patient we recorded survival outcomes (alive or dead) in the corresponding intervals of time for the four examined survival scores. For each outcome and score we built a specific logistic model predicting the probability of survival. Specificity and sensitivity were calculated by using varying cut-off points from zero (all predicted dead) to one (all predicted alive). For each decision probability we recorded the one-specificity versus sensitivity on the ROC curve. The estimated AUC computed by the trapezoid rule is the criterion for score performance for predicting the outcome. We compared logistic model performance of the four scores for a specific outcome, by comparing the AUC of the ROC curves. ROC comparisons for the four scores, for a specific outcome, were performed by using a contrast matrix. We used the MEWS curve as the reference curve for comparisons.

We created a new score for predicting mortality utilizing the classical "sepsis stages" (i.e. sepsis, severe sepsis, and septic shock). We divided our patients into four groups with ascending severity: 1 = SIRS; 2 = sepsis; 3 = severe sepsis; 4 = septic shock. We modeled by logistic regression the log odds for dying after a specific period (one day, five days, in hospital, etc). Sepsis stage was used as the categorical explanatory (independent) variable. The solution of the model is the weight for each category of sepsis group, while the categories are not equally departed from each other. The weights for each model are the best possible weights (minimal Log likelihood) for our sample of patients. Thereafter, we calculated the AUC of the ROC curve for its predictive value, and compared it with the four scores presented in our work. The curve comparison was based on the method proposed by DeLong et al. [19]. Predictive accuracy for each model was assessed by comparing the observed and the expected mortality in different intervals of time by using the Hosmer-Lemeshow (HL) goodness-of-fit test. The chi square test was carried out to determine if the observed and expected frequencies are significantly different. A *P *value greater than 0.05 for the HL test is considered suggestive of a calibrated model.

For our calculations we used SAS 9.2 software (Procedure univariate freq and logistic (SAS, Cary, NC, USA)) [20].

The study was approved by the Carmel Hospital Institutional Review Board. The need for informed consent was waived.

## Results

### Study population

During the 15-month study period, 1,072 patients admitted to general internal medicine departments met the criteria of sepsis on their admission. The mean age of our study group was 74.7 ± 16.1 years, with 49% of the study population being over 80 years of age, and 11.7% over 90 years of age. Male to female ratio was 1.08:1. Most of the study population (96.2%) was admitted through the emergency department, with the rest being transferred from other departments in the hospital. At admission, 5% of our study cohort fulfilled the criteria for septic shock, and 9.3% for severe sepsis. Seventy-seven patients (7.2%) were transferred to other departments, including 19 patients (1.8%) transferred to the ICU. Suspected source of infection on admission was as follows: pneumonia (43%), urinary tract infection (27%), unknown source (14%), skin and soft tissue infections (6.3%), and other source (9%). Data for 17 patients (1.5%) were missing.

### Outcomes

The mean time to last follow up was 90 ± 62 days. During this follow-up period, 387 patients died with an overall mortality rate of 36.1%. The 28-day in-hospital mortality was 19.5%. The overall in-hospital mortality was 21.9%. Mortality rates were found to be 4%, 11.2%, 15.6%, 24.9%, and 30.4% for 1, 5, 10, 30, and 60 days after admission. Mean time to death was 30 days (range: 0 to 290) following admission. Length of stay was 8.77 (range: 1 to 76) days.

### Scoring systems

Table 2 compares the characteristics and calculated clinical scores of survivors versus non-survivors. Our study group was heterogeneous and included patients with different degrees of severity according to the examined scores [see Figures S1 to S4 in Additional file 1]. We had patients with low, mid-range, and high values of the calculated scores. In comparison with the original studies [14-17], we had a higher ratio of patients with high scores (MEWS >4: 33% vs. 10% [14], MEDS >12: 11.4% vs. 7% [16], REMS >15: 11.2% vs. 0.8% [17], SCS >7: 23.6% to 25.6% vs. 1.7% [15]).

**Table 2:** Comparison between survivors and non-survivors

	Dead	Alive	*P *value
**Age (Mean ± SD)**	81.50 ± 10.02	70.83 ± 17.61	<0.0001
**Female (%)**	45.7	48.9	0.31
**Male (%)**	54.2	51.1	
**Diabetes mellitus (%)**	41.4	33.7	0.012
**Long-term care facility residents (%)**	30.5	14.5	<0.0001
**Debilitated patients (%)**	77	37	<0.0001
**Terminal illness (%)**	20.6	6.3	<0.0001
**MEDS (Mean ± SD)**	7.7 ± 3.0	4.9 ± 3.0	<0.0001
**REMS (Mean ± SD)**	11.9 ± 4.6	8.4 ± 3.9	<0.0001
**MEWS (Mean ± SD)**	4.5 ± 2.7	3.2 ± 2.1	<0.0001
**SCS (Mean ± SD)**	14.9 ± 4.0	11.3 ± 3.5	<0.0001

MEDS, mortality in emergency medicine sepsis score; MEWS, modified early warning score; REMS, rapid emergency medicine score; SCS, simple clinical score; SD, standard deviation

The averages and medians of the examined scores for patients who died during the first five days and those who survived this period of time were found to be significantly different [see Figures S1 to S4 in Additional file 1]. These significant differences were found also at the end of the follow-up period (Table 2).

**Figure 1:**
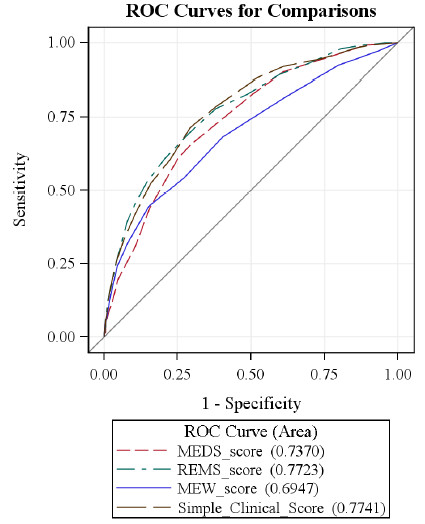
**ROC curves for overall in-hospital mortality of the examined scoring systems**. MEDS, mortality in emergency medicine sepsis score; MEW, modified early warning; REMS, rapid emergency medicine score; ROC, receiver operator characteristic.

The ROC curves for in-hospital mortality are shown in Figure 1. The AUC values and HL *P *values for mortality at different time points for the four scoring systems are summarized in Table 3. The five days in-hospital mortality was predicated with acceptable values of AUC by the MEDS, REMS, and SCS scores (0.77 to 0.8), The 28-day in-hospital mortality was predicted with acceptable value of AUC (0.79) by the REMS and SCS. The MEWS had the least reliable values of AUC. The differences of the AUCs of the MEDS, REMS, and SCS compared with MEWS were found to be significant with *P *values of less than 0.05. Of notice, all the scoring systems predicted 1 to 10 days mortality better than mortality after 30-60 days. The "sepsis stages" score was found to have an AUC of 0.65 for predicting in-hospital mortality, which is lower than the AUC of each of the four scoring systems.

**Table 3:** The discrimination (AUC) and calibration (Hosmer-Lemeshow goodness-of-fit) power of the examined scoring systems in different intervals of time

		1-day mortality	5-day mortality	10-day mortality	30-day mortality	60-day mortality	28-day in-hospital mortality	Overall in-hospital mortality
**MEDS**	**AUC (95% CI)**	0.79 (0.73-0.85)	0.77 (0.73-0.81)	0.79 (0.76-0.83)	0.75 (0.71-0.78)	0.74 (0.71-0.77)	0.75 (0.71-0.78)	0.73 (0.70-0.77)
	**HL - *P *value**	0.19	0.0001	0.06	0.02	0.02	0.02	0.29
**MEWS**	**AUC (95% CI)**	0.83 (0.77-0.88)	0.73 (0.68-0.78)	0.72 (0.68-0.77)	0.67 (0.63-0.71)	0.65 (0.62-0.69)	0.70 (0.66-0.74)	0.69 (0.65-0.73)
	**HL - *P *value**	0.57	0.31	0.28	0.86	0.73	0.43	0.32
**REMS**	**AUC (95% CI)**	0.87 (0.83-0.92)	0.80 (0.76-0.84)	0.80 (0.77-0.84)	0.76 (0.72-0.79)	0.74 (0.71-0.77)	0.79 (0.75-0.81)	0.77 (0.73-0.80)
	**HL - *P *value**	0.85	0.29	0.44	0.21	0.22	0.26	0.75
**SCS**	**AUC (95% CI)**	0.85 (0.80-0.90)	0.79 (0.76-0.83)	0.80 (0.77-0.84)	0.77 (0.74-0.81)	0.76 (0.73-0.79)	0.79 (0.75-0.82)	0.77 (0.74-0.80)
	**HL - *P *value**	0.49	0.14	0.68	0.54	0.79	0.16	0.71

AUC, area under the curve; CI, confidence interval; HL, Hosmer-Lemeshow; MEDS, mortality in emergency medicine sepsis score; MEWS, modified early warning score; REMS, rapid emergency medicine score; SCS, simple clinical score.

The HL goodness-of-fit test results are shown in Table 3. REMS. SCS and MEWS scoring systems were found to be accurate predictors of mortality with good calibration. The MEDS score was inaccurate in its predictive value for mortalities, as reflected by the low rates of *P *value.

## Discussion

The present study compared the ability of four disease-severity scoring systems to predict mortality in a growing group of patients, that is, septic patients admitted to general internal medicine departments. Previous studies investigating scoring systems in septic patients were largely confined to ICU settings [8-12], whereas today the majority of septic patients are actually admitted to general medicine departments [4,5]. Evaluation of disease severity and prognosis for this group of patients as well as clinical trials investigating new therapies and evaluating performance require appropriate disease-severity scoring system for these patients.

Simple categorical description of this group of patients using the classical definition of sepsis stages is not satisfactory as can be seen from the low AUC value (0.65) for predicting in-hospital mortality. Our results indicate that all scoring systems tested in our study are better predictors of in-hospital mortality.

When considering both the discrimination (AUC) and calibration (HL goodness-of-fit) power of the examined scoring systems, our study shows that SCS and REMS are appropriate mortality prediction models for patients with sepsis admitted to general internal medicine departments, for all of the examined time points.

The scoring systems evaluated in the present study are simple and based on available clinical and laboratory parameters, as opposed to the widely used, but complicated, scoring systems commonly used for ICU patients. These systems utilize parameters that are frequently unavailable in a general medical department (e.g., continuous urine output monitoring, and partial pressure of arterial oxygen).

The MEWS, SCS, and REMS scores were developed in a general group of patients with low mortality rates (7.9%, 4.7%, and 2.4%, respectively) [14,15,17]. In the present study, which was confined to a disease-specific group of patients, the scores lost some of their predictive strength (AUC of 0.85 to 0.90, and 0.85 for SCS and REMS, respectively [15,17]). The MEWS had a low AUC in the original study [14].

The MEDS is the only scoring system that was developed in a similar but not identical group of patients, that is "patients at increased risk for infection" [16]. Such patients were included in the original study if blood cultures were drawn from them upon admission by order of the attending physician. Almost 45% of the patients in the original study did not meet the criteria of SIRS. This can explain the low mortality rate in the original study (5.3 to 5.7%) compared with our study group (19.5%). This may also underlie the difference from our findings and the fact that in our study of patients with SIRS this score had a lower correlation to 28-day mortality (AUC of 0.75 compared with 0.82 in the original study).

Another significant difference between our study and the original studies that derived and validated the examined scoring systems is the higher mean of age (74.7 years compared with 62, 62, 63, and 61.8 years for SCS, REMS, MEWS, and MEDS, respectively). As the Western population ages, the epidemiology of sepsis is changing. The high age mean in the present study reflects this change, and may partially account for the higher mortality rate in our study group compared with the original studies that assessed the four scoring systems. It has been shown that the average age of patients with sepsis increased constantly over time from 57.4 years between 1979 and 1984 to 60.8 years between 1995 and 2000 [1]. As life expectancy increases every year, it is not surprising that the average age of septic patients has increased even further in the past decade [2]. Other recently published studies support this fact. In a prospective study conducted in Spain in 2003, the mean age of septic patients admitted to different hospital departments, including ICUs, was 69 years [5]. It is known that increasing age is a major contributing factor for mortality from sepsis, regardless of co-morbidities [3]. This fact emphasizes the need for including elderly patients in clinical trails investigating new therapies. This fact may explain the poor performance of the MEWS as the only score examined in our study that did not include age. Age was significantly and substantially different between survivors and non-survivors in our study population (mean of 70.8 years compared with 81.5 years, respectively).

An important point that emerges from our data is the different mortality rates at different points of time. Most studies utilize the overall in-hospital mortality or 28-day in-hospital mortality as endpoints. Our data indicate that these outcomes are significantly lower than the overall mortality within a corresponding period of time (e.g. 28-day in-hospital mortality of 19.5% compared with 30-day overall mortality of 24.9%). Thus, the 28-day in-hospital mortality may fail to capture the true impact of sepsis on subsequent outcomes, and may be too insensitive, failing to capture important effects on surrogate outcomes, such as the effects of potential therapies [21].

The fact that in our study a large proportion of deaths occurred later after admission (30% within 60 days compared with 16% within 10 days) may reflect the fact that the current septic population in general internal medicine departments is elderly, has numerous chronic underlying diseases, and takes multiple prescription drugs. These patients are frequently afflicted by severe deconditioning upon admission and are prone to in-hospital complications. Thus, the attributable mortality of sepsis *per se *may be lower than the high rates observed in our study.

Of interest is the fact that the examined scoring systems predict short-term mortality better than long-term mortality. This fact demonstrates the ability of these scoring systems to identify patients with sepsis at risk for immediate deterioration. The differences are more remarkable for MEWS and REMS, which are based on physiological parameters on admission, including neurological examination, rather than basic functional and mental status or chronic diseases, which appear to contribute to late mortality. For early death (presented as five-days mortality) all examined scoring systems were found to be significantly lower for patients who survived compared with those who died [see Figures S1 to S4 in Additional file 1]. In this respect, these tools were found to be appropriate for detection of patients at immediate risk, and thus can lead to intensified diagnostic and treatment approaches.

To the best of our knowledge, this study is the first to compare the performance of different scoring systems for septic patients admitted to general internal medicine departments. Our study has the strength of being prospective, and adhering to the widely used and accepted definition of sepsis. However, some limitations should be noted. Our study population was confined to a single center. The study included patients admitted with sepsis, but not those who developed sepsis during their hospitalization or patients that did not fit criteria of SIRS. Furthermore, the scoring systems were calculated on admission only.

## Conclusions

The present study shows that two of the examined scoring systems, REMS and SCS, can predict mortality in septic patients admitted to general internal medicine departments with good accuracy, and can thus be utilized in this enlarging clinical setup.

## Key messages

• Sepsis is a common medical issue with increasing incidence and high rate of mortality (30-day mortality of 25% in our study group).

• Most of septic patients are admitted to general internal medicine departments.

• From the examined scoring systems in our study, SCS and REMS were found to predict outcomes in septic patients admitted to general internal medicine departments with good accuracy.

## Abbreviations

ACCP, American College of Chest Physicians; AUC, area under curve; AVPU score, alert, responding to voice, responding to pain, unconscious score; EMR, electronic medical record; MEDS, mortality in emergency department sepsis score; MEWS, modified early warning score; REMS, rapid emergency medicine score; ROC, receiver operating characteristics; SCCM, Society of Critical Care Medicine; SCS, simple clinical score; SIRS, systemic inflammatory response syndrome; SSC, surviving sepsis campaign.

## Competing interests

The authors declare that they have no competing interests.

## Authors' contributions

NGZ participated in the design of the study, supervised the process of data collection and analysis, interpretation, and prepared the manuscript for publication. MV helped in data management, format and analysis, and in preparing the manuscript for publication. AL contributed to the design of the study and performed the statistical analysis. GW contributed to the design of the study, data analysis, and preparing the manuscript for publication. HB participated in the design of the study, the process of data analysis and interpretation, and helped in preparing the manuscript for publication.

## Supplementary Material

Additional file 1 **Supplementary figures S1 to 4**. Figure S1: The distribution of mortality in emergency medicine sepsis score (MEDS) for patients who survived (upper diagram) and patients who died (lower diagram) during the first five days of hospitalization. Figure S2: The distribution of rapid emergency medicine score (REMS) for patients who survived (upper diagram) and patients who died (lower diagram) during the first five days of hospitalization. Figure S3: The distribution of modified early warning score (MEWS) for patients who survived (upper diagram) and patients who died (lower diagram) during the first five days of hospitalization. Figure S4: The distribution of simple clinical score (SCS) for patients who survived (upper diagram) and patients who died (lower diagram) during the first five days of hospitalization. Click here for file
