# Supplementary material for: Assessment of disease-severity scoring systems for patients with sepsis in general internal medicine departments
Source: Crit Care. 2011 Mar 14;15(2):R95. doi: 10.1186/cc10102 (PMC3219360; doi:10.1186/cc10102)
Supplement: Additional file 1 — Supplementary figures S1 to 4. Figure S1: The distribution of mortality in emergency medicine sepsis score (MEDS) for patients who survived (upper diagram) and patients who died (lower diagram) during the first five days of hospitalization. Figure S2: The distribution of rapid emergency medicine score (REMS) for patients who survived (upper diagram) and patients who died (lower diagram) during the first five days of hospitalization. Figure S3: The distribution of modified early warning score (MEWS) for patients who survived (upper diagram) and patients who died (lower diagram) during the first five days of hospitalization. Figure S4: The distribution of simple clinical score (SCS) for patients who survived (upper diagram) and patients who died (lower diagram) during the first five days of hospitalization. [file cc10102-S1.DOC]

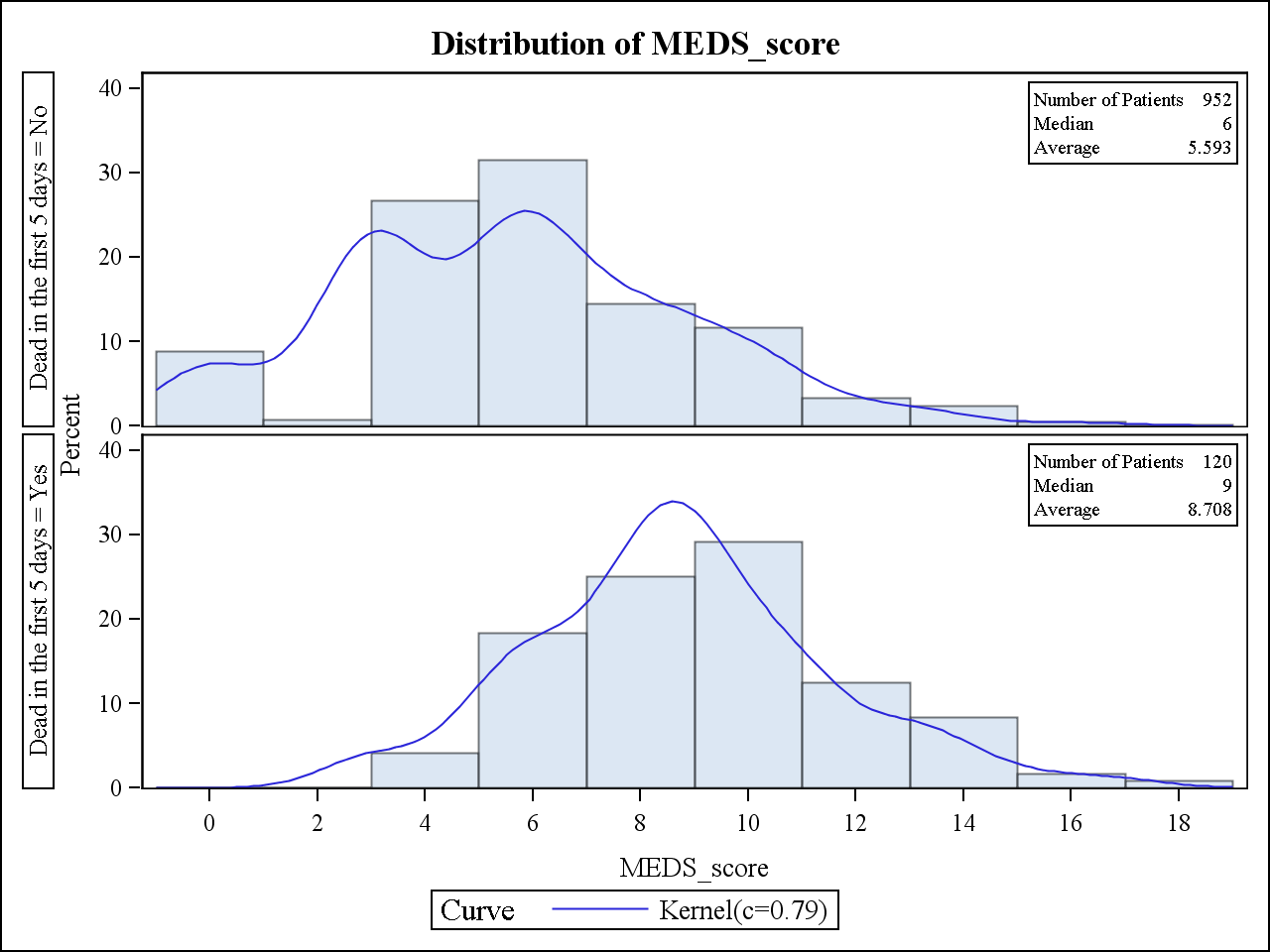


P<0.001

**Figure S1**-The distribution of MEDS score for patients who survived (upper diagram) and patients who died (lower diagram) the first 5 days of hospitalization.


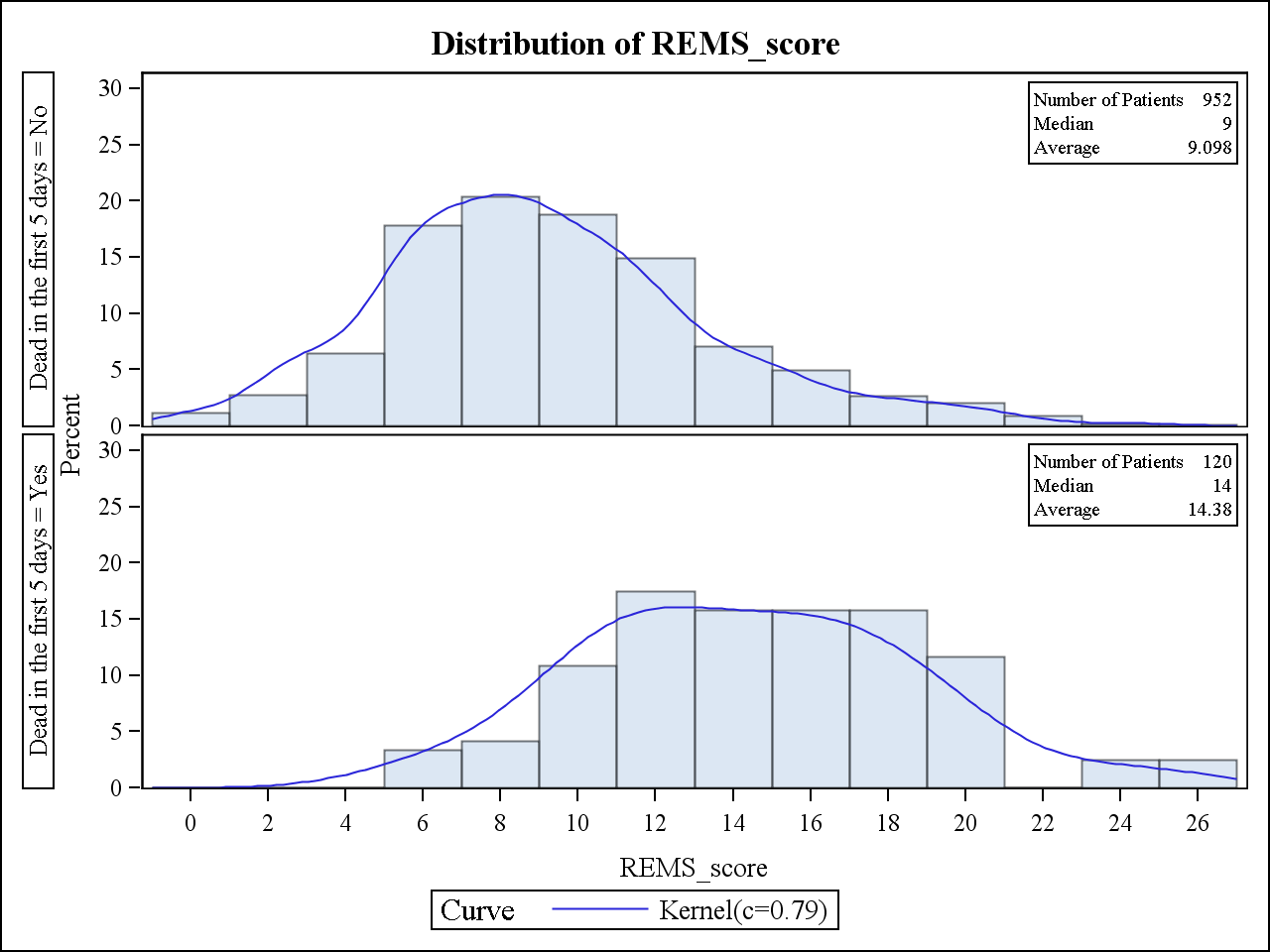


P<0.001

**Figure S2**-The distribution of REMS for patients who survived (upper diagram) and patients who died (lower diagram) the first 5 days of hospitalization.


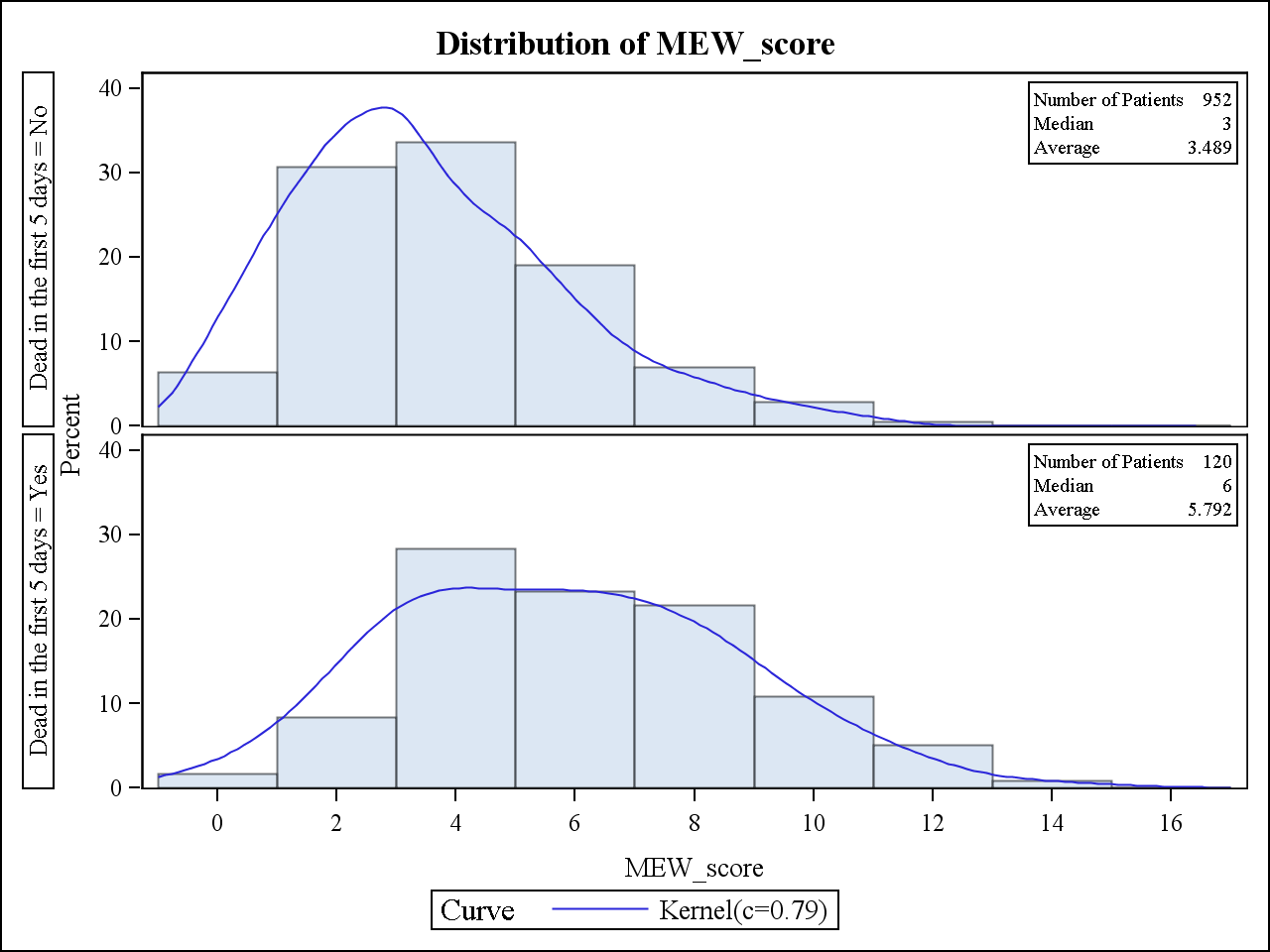


P<0.001

**Figure S3-** The distribution of MEWS for patients who survived (upper diagram) and patients who died (lower diagram) the first 5 days of hospitalization.


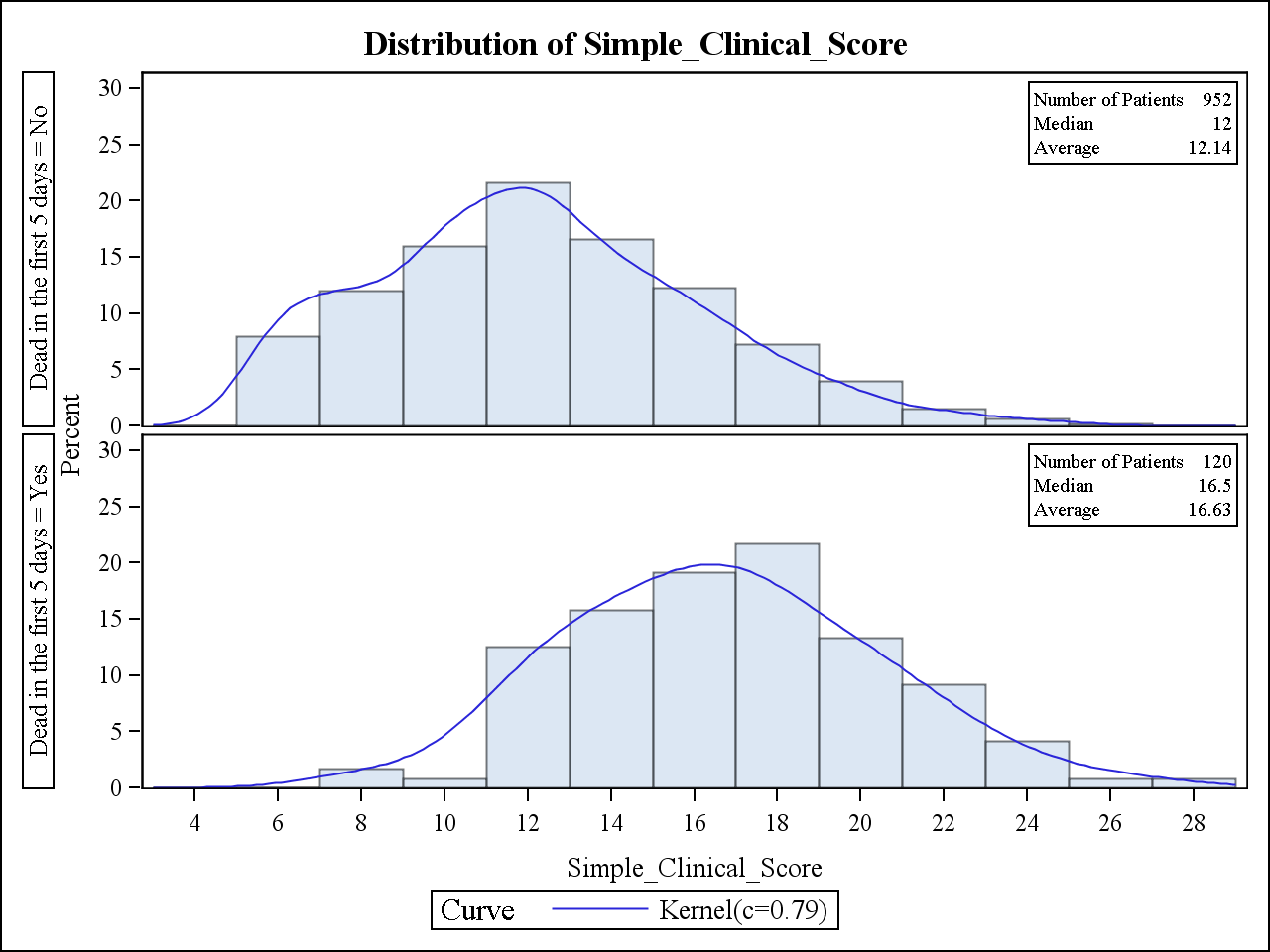


P<0.001

**Figure S4**- The distribution of Simple Clinical Score for patients who survived (upper diagram) and patients who died (lower diagram) the first 5 days of hospitalization.
